# Five years of exercise intervention at different intensities and development of white matter hyperintensities in community dwelling older adults, a Generation 100 sub-study

**DOI:** 10.18632/aging.203843

**Published:** 2022-01-18

**Authors:** Anette Arild, Torgil Vangberg, Hanne Nikkels, Stian Lydersen, Ulrik Wisløff, Dorthe Stensvold, Asta K. Håberg

**Affiliations:** 1 Department of Neuromedicine and Movement Science, Faculty of Medicine and Health Sciences, NTNU Norwegian University of Science and Technology, Trondheim, Norway; 2 Department of Clinical Medicine, Faculty of Health Sciences, UiT The Arctic University of Norway, Tromsø, Norway; 3 PET Center, University Hospital of North Norway, Tromsø, Norway; 4 Department of Radiology and Nuclear Medicine, St. Olavs Hospital, Trondheim University Hospital, Trondheim, Norway; 5 Department of Mental Health, Faculty of Medicine and Health Sciences, NTNU Norwegian University of Science and Technology, Trondheim, Norway; 6 Department of Circulation and Medical Imaging, Faculty of Medicine and Health Sciences, NTNU Norwegian University of Science and Technology, Trondheim, Norway; 7 Department of Cardiology, St. Olavs Hospital, Trondheim University Hospital, Trondheim, Norway

**Keywords:** leukoaraiosis, senior, physical fitness, prospective, randomized controlled trial

## Abstract

We investigated if a five-year supervised exercise intervention with moderate-intensity continuous training (MICT) or high-intensity interval training (HIIT) versus control; physical activity according to national guidelines, attenuated the growth of white matter hyperintensities (WMH). We hypothesized that supervised exercise, in particular HIIT, reduced WMH growth. Older adults from the general population participating in the RCT Generation 100 Study were scanned at 3T MRI at baseline (age 70–77), and after 1-, 3- and 5-years. At each follow-up, cardiorespiratory fitness was measured with ergospirometry, and physical activity plus clinical data collected. Manually delineated total WMH, periventricular (PWMH), deep (DWMH), and automated total white matter hypointensity volumes were obtained. No group by time interactions were present in linear mixed model analyses with the different WMH measurements as outcomes. In the combined exercise (MICT&HIIT) group, a significant group by time interaction was uncovered for PWMH volume, with a larger increase in the MICT&HIIT group. Cardiorespiratory fitness at the follow-ups or change in cardiorespiratory fitness over time were not associated with any WMH measure. Contrary to our hypothesis, taking part in MICT or HIIT over a five-year period did not attenuate WMH growth compared to being in a control group following national physical activity guidelines.

## INTRODUCTION

White matter hyperintensities (WMH), also known as leukoaraiosis, are the most common finding on brain MRI in persons aged 50 and older [1, 2]. Its presence is associated with functional impairments such as gait disturbance [3, 4] and cognitive decline [5], as well as diseases such as depression [6], stroke and dementia [7]. WMH appear as hyperintense areas in the brain on T2-weighted MRI scans, e.g., fluid-attenuated inversion recovery (FLAIR), and are usually symmetrically distributed around the ventricles (periventricular WMH, PWMH) and in the deep white matter (deep WMH, DWMH). PWMH and DWMH are associated with overlapping as well as unique genetic markers [8], etiologies [9, 10], and clinical correlates [11, 12], leading to the two subdivisions being regarded as different entities [13]. Even though WMH have a clinical impact in old age and several risk factors are identified [14–16], there is no consensus as to how to treat or reduce them.

Observational studies suggest that physical activity and exercise may limit WMH. Two systematic reviews found physical activity to be associated with less WMH [17, 18], and the effect appeared particularly prominent in older adults [19, 20]. Both participating in physical activity and aerobic exercise training can improve cardiorespiratory fitness, which is suggested as a central mechanism for the effect of training on the brain [21]. Still, three recent intervention studies did not uncover a positive effect of 6–24 months of physical activity, aerobic exercise or multimodal lifestyle intervention compared to usual care or general life-style advice [22–24]. Intervention studies with more intense exercise aimed at maximizing cardiovascular fitness, longer intervention periods with several follow-ups to monitor change over time in both WMH and fitness, and objective measures of cardiovascular fitness, i.e., VO_2peak_ measured during ergospirometry, are suggested to resolve the current conundrum [17, 25, 26]. Since physical activity appears to limit WMH growth most effectively in older adults, this group is a highly relevant target group for exercise intervention studies.

In a sub-study of the randomized controlled trial (RCT) Generation 100 Study in adults born between 1936 and 1942 [27, 28], we investigated the evolution of WMH on brain MRI acquired at 3T at baseline, and after one, three, and five years of supervised exercise with either moderate-intensity continuous training (MICT) or high-intensity interval training (HIIT) compared to a control group that followed the Norwegian national recommendations for physical activity.

We hypothesized that manually derived WMH volume would grow less in the supervised training groups, particularly in the HIIT group, compared to the control group, and that a greater gain in VO_2peak_ would translate to attenuated WMH growth. Since there is increased use of automated methods for WMH delineation, analyses with both the gold standard manual and an automated method were performed.

## RESULTS

### Participants and participation

**Figure 1:**
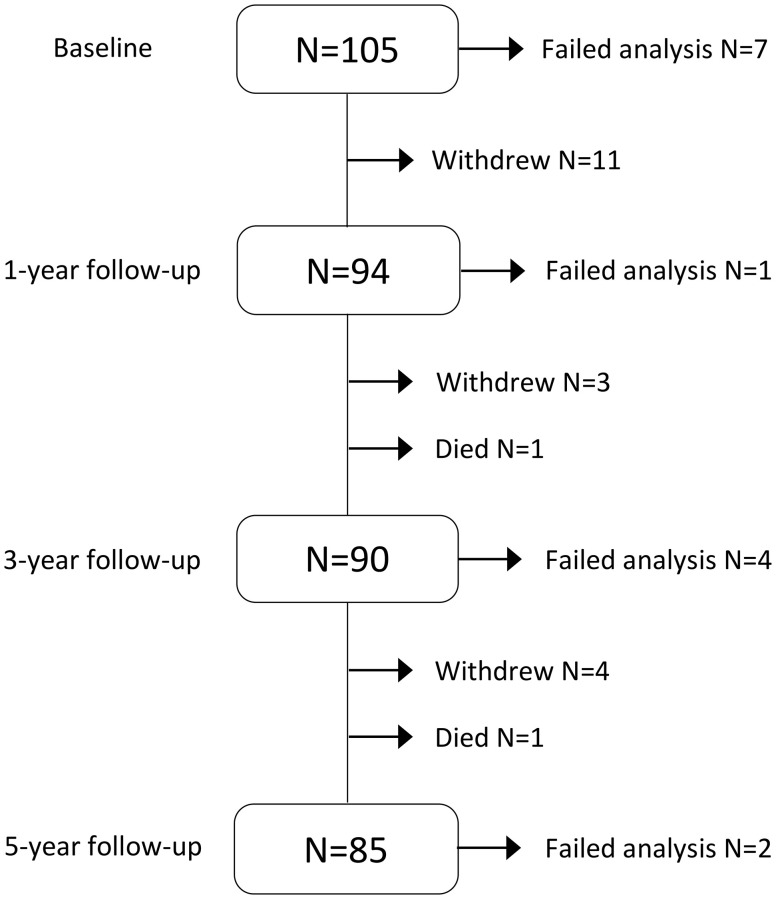
**Flowchart of participation, attrition and MRI data included in statistical analysis during the five-year intervention.** Failed analysis included corrupted images, motion or other artifacts interfering with manual delineation and/or automated analysis (Figures 3 and 4).

In total, 105 MRI participants were included and scanned at baseline. After five years, 85 participants remained in the study, none of whom were diagnosed with MCI or dementia. Participants who withdrew from the study, did so mainly during the first year. Two participants died of cancer in the HIIT group during the study. See Figure 1 for an overview of participants at each timepoint and MRI scans passing quality assessment and included in statistical analysis. For variables used in this paper, data were missing completely at random (*X*^2^ (1737) = 1747.66, *p* = 0.42).

The results of the baseline comparisons of demographic and clinical characteristics of those participating and not participating in the MRI sub-study are displayed in Table 1. Participants participating in the MRI sub-study had a higher level of education, VO_2peak_ was on average 1.7 mL/kg/min higher, and Hospital Anxiety and Depression Scale (HADS) scores were lower than in the group not scanned. There were also minimal though significant differences in age and blood fat values, with the participants with MRI having lower triglycerides (median 0.93 vs. 1.01 mmol/L) and higher HDL (median 1.74 vs. 1.66 mmol/L) levels.

**Table 1:** Demographic and clinical characteristics at baseline of participants in the RCT Generation 100 (G100) study taking and not taking part in the neuroimaging study.

	**G100 without MRI (*N* = 1461)**	**G100 with MRI (*N* = 105)**	***p*-value**
Age^a^ (years)	72.6 (3.2)	72.2 (2.6)	**0.045**
Women^b^ (%)	50.5	49.5	0.845
Education^a^ (%)			**0.003**
Primary school	15.3	8.7	
High school	35.2	26.9	
University	49.5	64.4	
Living alone^b^ (%)	24.8	29.8	0.253
Current smoker^b^ (%)	8.5	8.7	0.944
Pack years^a,c^	13.7 (18)	11.3 (17)	0.246
Hypertension^b,d^ (%)	55.9	49.0	0.179
Diabetes^b,e^ (%)	8.6	4.7	0.160
Waist circumference^f^ (cm)	94.2 (11.1)	93.7 (10.6)	0.639
Body mass index^f^ (kg/m^2^)	26.0 (3.6)	25.9 (3.3)	0.848
Triglycerides^a^ (mmol/L)	1.0 (0.6)	0.9 (0.5)	**0.032**
LDL^a^ (mmol/L)	3.4 (1.4)	3.3 (1.5)	0.621
HDL^a^ (mmol/L)	1.7 (0.7)	1.7 (0.7)	**0.043**
Total cholesterol^f^ (mmol/L)	5.6 (1.1)	5.8 (1.0)	0.246
hsCRP (mg/L)^a^	1.3 (1.8)	1.2 (1.6)	0.379
VO_2peak_^f^ (mL/kg/min)	28.6 (6.4)	30.3 (6.4)	**0.012**
Testing on bike^g^ (%)	3.0	2.9	1.000
Reached VO_2max_^b^ (%)	59.9	65.4	0.269
HADS^f^ (total score)	6.2 (4.4)	4.6 (3.6)	**<0.001**

^a^Mann-Whitney *U*-test (percentage or median and interquartile range (IQR)); ^b^Chi-square (percentage); ^c^Pack year: 20 cigarettes daily for one year; ^d^Hypertension: SBP ≥140 mmHg and/or DBP ≥90 mmHg and/or use of blood pressure medication; ^e^Diabetes: fasting p-glucose ≥7.0 mmol/L and/or HbA1c ≥48 mmol/mol and/or diagnosed (type 1 or type 2). ^f^Independent *t*-test (mean and standard deviation (SD)); ^g^Fischer’s exact (percentage). Abbreviations: G100: Generation 100; LDL: low-density lipoprotein; HDL: high-density lipoprotein; hsCRP: high-sensitive C-reactive protein; SBP: systolic blood pressure; DBP: diastolic blood pressure; HbA1c: glycosylated hemoglobin; VO_2peak_: peak oxygen uptake, a combination of VO_2max_ and VO_2peak_ values; VO_2max_: maximal oxygen uptake; HADS: Hospital Anxiety and Depression Scale.

The baseline and the five-year demographic, clinical and VO_2peak_ characteristics of the control, MICT and HIIT group participants in the MRI study are shown in Table 2. There were no differences between the three groups at baseline or after five years (Table 2A and 2B).

**Table 2:** Comparison of the control, moderate-intensity continuous training (MICT) and high-intensity interval training (HIIT) exercise groups at baseline (A) and after five years (B).

**A. Baseline characteristics**				
	**Control (*N* = 48)**	**MICT (*N* = 24)**	**HIIT (*N* = 33)**	***p*-value**
Age^a^ (years)	72.3 (2.4)	71.8 (2.2)	72.3 (3.1)	0.791
Women^b^ (%)	52.1	54.2	42.4	0.607
Education^b^ (%)				0.501
Primary school	8.3	12.5	6.3	
High school	33.3	20.8	21.9	
University	58.3	66.7	71.9	
Living situation^b^ (%)				0.953
Alone	31.3	29.2	28.1	
With partner	68.8	70.8	71.9	
Current smoker^c^ (%)	10.4	4.3	9.4	0.817
Pack years^a,d^	9.8 (11)	18.5 (40)	13.8 (23)	0.330
Hypertension^b,e^ (%)	50.0	47.8	45.2	0.915
Diabetes^c,f^ (%)	2.1	8.7	6.5	0.432
Waist circumference^g^ (cm)	93.3 (10.9)	93.5 (9.2)	94.4 (11.3)	0.897
Body mass index^g^ (kg/m^2^)	25.9 (3.3)	25.9 (3.4)	26.1 (3.3)	0.956
Triglycerides^a^ (mmol/L)	0.9 (0.4)	0.9 (0.4)	0.9 (0.5)	0.958
LDL^a^ (mmol/L)	3.5 (1.5)	3.2 (1.1)	3.3 (1.6)	0.223
HDL^a^ (mmol/L)	1.7 (0.7)	1.7 (0.7)	1.8 (0.8)	0.917
Total cholesterol^a^ (mmol/L)	6.1 (1.7)	5.6 (0.9)	5.9 (1.4)	0.145
hsCRP^a^ (mg/L)	1.1 (1.4)	1.0 (1.2)	1.5 (1.6)	0.431
VO_2peak_^g^ (mL/kg/min)	30.3 (6.5)	30.0 (5.7)	30.4 (6.9)	0.971
Testing on a bike^c^ (%)	2.1	0.0	6.1	0.454
Reached VO_2max_^b^ (%)	64.6	52.2	75.8	0.187
HADS^a^ (total score)	4 (5)	4 (5)	4 (5)	0.953
**B. Follow up characteristics at five years**				
Age^a^ (years)	76.8 (2.2)	77.2 (1.9)	77.4 (3.2)	0.841
Women^b^ (%)	48.6	52.4	44.8	0.869
Education^b^ (%)				0.317
Primary school	2.9	14.3	7.1	
High school	31.4	14.3	17.9	
University	65.7	71.4	75.0	
Living situation^b^ (%)				0.525
Alone	35.3	21.1	26.9	
With partner	64.7	78.9	73.1	
Current smoker^c^ (%)	6.3	5.3	0.0	0.467
Hypertension^b,e^ (%)	64.5	44.4	52.0	0.362
Diabetes^c,f^ (%)	0.0	11.8	8.7	0.110
Waist circumference^g^ (cm)	94.2 (12.9)	94.8 (8.9)	94.7 (11.1)	0.975
Body mass index^g^ (kg/m^2^)	25.9 (3.8)	26.0 (3.6)	26.0 (2.5)	0.988
Triglycerides^a^ (mmol/L)	0.9 (0.5)	0.8 (0.4)	1.0 (0.6)	0.261
LDL^g^ (mmol/L)	3.4 (1.0)	2.9 (1.0)	3.1 (0.8)	0.152
HDL^a^ (mmol/L)	1.6 (0.7)	1.6 (0.6)	1.8 (0.6)	0.724
Total cholesterol^g^ (mmol/L)	5.6 (1.1)	5.0 (1.1)	5.5 (1.0)	0.115
hsCRP^a^ (mg/L)	1.4 (2.2)	1.1 (1.3)	1.5 (1.7)	0.364
VO_2peak_^g^ (mL/kg/min)	30.1 (7.6)	28.6 (5.3)	30.5 (6.1)	0.633
Testing on a bike^c^ (%)	2.7	10.5	8.0	0.506
Reached VO_2max_^b^ (%)	70.3	52.6	68.0	0.400
HADS^a^ (total score)	5 (6)	5 (5)	3 (5)	0.062
MoCA^a^ (total score)	26 (4)	27 (3)	27 (3)	0.270

^a^Kruskal-Wallis H test (median and IQR); ^b^Chi-square (percentage); ^c^Fischer’s exact (percentage); ^d^Pack year: 20 cigarettes daily for one year; ^e^Hypertension: SBP ≥140 mmHg and/or DBP ≥90 mmHg and/or use of blood pressure medication; ^f^Diabetes: fasting p-glucose ≥7.0 mmol/L and/or HbA1c ≥48 mmol/mol and/or diagnosed (type 1 or type 2). ^g^One-way ANOVA (mean and SD). Abbreviations: MICT: moderate-intensity continuous training; HIIT: high-intensity interval training; LDL: low-density lipoprotein; HDL: high-density lipoprotein; hsCRP: high-sensitive C-reactive protein; SBP: systolic blood pressure; DBP: diastolic blood pressure; HbA1c: glycosylated hemoglobin; VO_2peak_: peak oxygen uptake, a combination of VO_2max_ and VO_2peak_ values; VO_2max_: maximal oxygen uptake; HADS: Hospital Anxiety and Depression Scale; MoCA: Montreal Cognitive Assessment scores, presented as raw scores.

During the supervised exercise sessions, the HIIT group trained on average at 88% of peak heart rate and at an intensity of 16.9 on the Borg scale, while the MICT group trained at 73% of peak heart rate with a mean intensity of 13.8 on the Borg scale.

**Figure 2:**
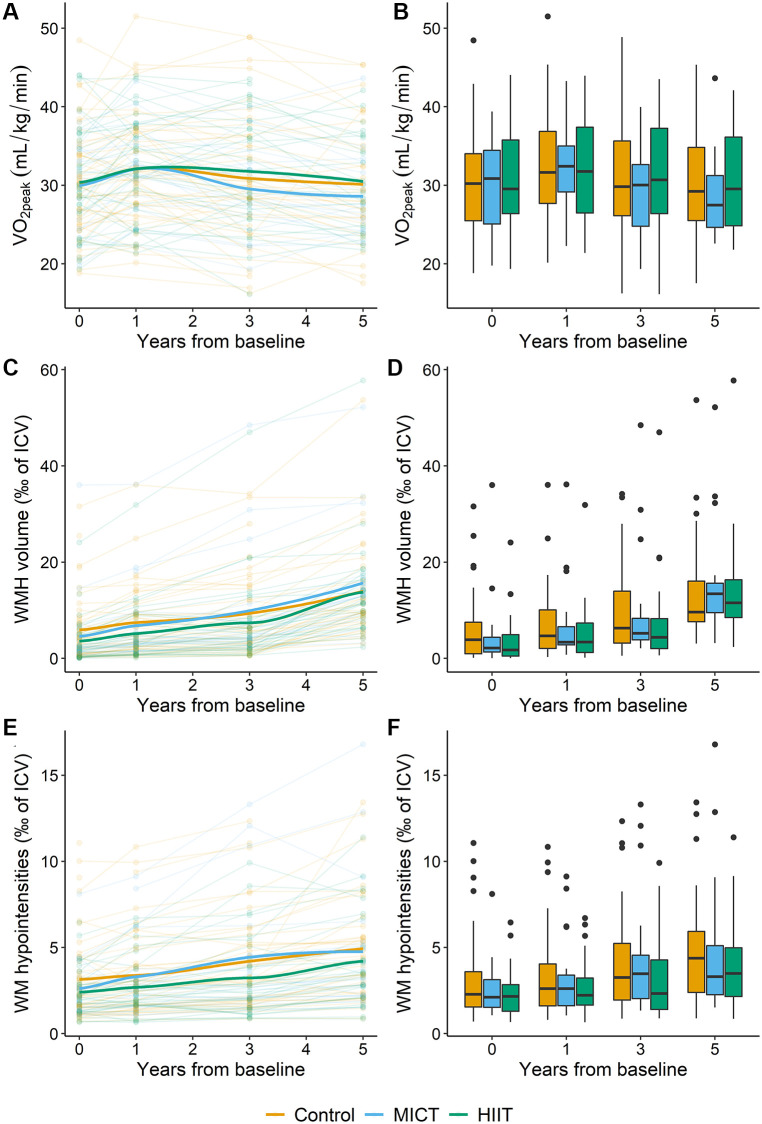
**Overview of the development of VO_2peak_, WMH and WM-hypointensity volumes (given as ‰ of ICV) in the control (orange), MICT (blue) and HIIT (green) groups during the five-year intervention period.** (**A**) Each line represents the VO_2peak_ values for an individual participant according to group adherence with the thicker lines representing the mean of each group. (**B**) Box plot of VO_2peak_ in the three intervention groups during the intervention period. (**C**) Each line represents the WMH volume as ‰ of ICV for an individual participant according to group adherence with the thicker lines representing the mean of each group. (**D**) Box plot of WMH volume as ‰ of ICV in the three intervention groups during the intervention. (**E**) Each line represents the WM-hypointensity volume as ‰ of ICV for an individual participant according to group adherence with the thicker lines representing the mean of each group. (**F**) Box plot of WM-hypointensity volume as ‰ of ICV in the three intervention groups during the intervention. In the box plots (**B**, **D**, and **F**), the box indicates the interquartile range with the top line of the box representing the third quartile and the bottom line representing the first quartile. The whiskers extend up to the minimum or maximum values, but no further than 1.5 times the interquartile range, and the black dots represent data beyond the end of the whiskers. Abbreviations: VO_2peak_: peak oxygen uptake; WMH: white matter hyperintensities; ICV: intracranial volume; WM: white matter; MICT: moderate-intensity continuous training; HIIT: high-intensity interval training.

During the intervention period, VO_2peak_ increased significantly and similarly in all groups from baseline to one-year follow-up (estimate: 2.3, 95% CI 1.1 to 3.4, *p*-value <0.001) (Figure 2), and then declined to baseline levels at the five-year follow-up (not significant). There was no effect of group or group*time interaction on VO_2peak_.

Based on the questionnaires, adherence to the allocated physical activity or exercise program was good, ranging between 71.4% and 94.3% across the groups throughout the intervention (Table 3). Furthermore, participants in the HIIT group exercised at a significantly higher intensity level than both MICT and controls at all timepoints during the five-year intervention period, but exercise frequency and session duration were similar across the three groups at all timepoints (Table 4). The types of activities varied between the control, MICT and HIIT groups, with the HIIT group biking more the first year, swimming more at year three, and training more at fitness centers at year five than the other groups (Table 5).

**Table 3:** Adherence to the allocated exercise program during the intervention period in the control, moderate-intensity continuous training (MICT), and high-intensity interval training (HIIT) groups.

**Time**	**Control**	**MICT**	**HIIT**	***p*-value^a^**
One-year follow-up	38/42 (90.5%)	16/21 (76.2%)	23/31 (74.2%)	0.136
Three-year follow-up	32/39 (82.1%)	15/21 (71.4%)	26/30 (86.7%)	0.379
Five-year follow-up	33/35 (94.3%)	18/21 (85.7%)	23/29 (79.3%)	0.184

Given as adhering/total number at each timepoint (percentage). ^a^Difference between the groups at each timepoint was tested with Fischer’s exact test. Abbreviations: MICT: moderate-intensity continuous training; HIIT: high-intensity interval training.

**Table 4:** Weekly exercise frequency, duration, and intensity in the control, moderate-intensity continuous training (MICT) and high-intensity interval training (HIIT) group.

	**Mean (SD)**			***p*-value**		
	**Control**	**MICT**	**HIIT**	***Control* vs. *MICT***	***MICT* vs. *HIIT***	***Control* vs. *HIIT***
***One-year follow-up***						
Exercise frequency^1^	3.0 (1.3)	2.8 (1.3)	3.3 (1.3)	NS	NS	NS
Exercise duration^2^	45.7 (14.4)	46.8 (8.2)	47.9 (9.6)	NS	NS	NS
Minutes/week exercise	140.2 (77.3)	132.3 (75.5)	157.5 (70.9)	NS	NS	NS
Exercise intensity^3^	13.8 (2.0)	13.6 (0.9)	15.2 (1.5)	0.267	**<0.001**	**<0.001**
***Three-year follow-up***						
Exercise frequency^1^	3.0 (1.7)	2.9 (1.2)	3.3 (1.4)	NS	NS	NS
Exercise duration^2^	46.1 (14.0)	49.0 (10.0)	47.5 (12.2)	NS	NS	NS
Minutes/week exercise	146.9 (86.7)	147.8 (53.8)	155.5 (72.5)	NS	NS	NS
Exercise intensity^3^	13.2 (2.6)	13.4 (0.9)	15.6 (1.3)	0.265	**<0.001**	**<0.001**
***Five-year follow-up***						
Exercise frequency^1^	3.3 (1.6)	2.8 (1.3)	3.2 (1.4)	NS	NS	NS
Exercise duration^2^	48.4 (14.5)	50.1 (10.0)	44.4 (13.1)	NS	NS	NS
Minutes/week exercise	168.3 (92.7)	141.1 (75.3)	138.5 (75.9)	NS	NS	NS
Exercise intensity^3^	13.4 (1.7)	12.5 (2.1)	15.0 (1.4)	0.118	**<0.001**	**<0.001**

Difference between the groups at each timepoint was tested with Kruskal-Wallis test and if significant tested with Dunn’s test post-hoc. ^1^sessions per week; ^2^minutes per session; ^3^Borg scale, range 6–20. Abbreviations: MICT: moderate-intensity continuous training; HIIT: high-intensity interval training; NS: not significant.

**Table 5:** Frequency of different types of physical activity in the control, moderate-intensity continuous training (MICT) and high-intensity interval training (HIIT) group at each follow-up during the five-year intervention.

	**Mean (SD)**			***p*-value**		
	**Control**	**MICT**	**HIIT**	***Control* vs. *MICT***	***MICT* vs. *HIIT***	***Control* vs. *HIIT***
***One-year follow-up***						
Walking^a^	2.34 (1.20)	2.47 (0.95)	2.43 (1.72)	NS	NS	NS
Biking	0.75 (0.93)	1.03 (2.18)	1.74 (2.09)	0.198	**0.008**	**0.029**
Swimming	0.27 (0.49)	0.21 (0.30)	0.51 (0.76)	NS	NS	NS
Skiing^b^	0.71 (1.08)	0.71 (1.00)	0.73 (0.92)	NS	NS	NS
Fitness center	0.99 (1.19)	0.96 (1.18)	1.47 (1.36)	NS	NS	NS
Organized sports	0.15 (0.39)	0.27 (0.49)	0.32 (0.59)	NS	NS	NS
Other activities	0.23 (0.66)	0.21 (0.39)	0.53 (0.82)	NS	NS	NS
***Three-year follow-up***						
Walking^a^	2.26 (1.26)	1.97 (1.36)	2.54 (1.73)	NS	NS	NS
Biking	0.77 (1.16)	1.01 (2.01)	1.54 (1.91)	NS	NS	NS
Swimming	0.28 (0.60)	0.09 (0.12)	0.53 (0.66)	0.097	**0.004**	**<0.001**
Skiing^b^	0.68 (1.10)	0.87 (1.70)	0.72 (0.97)	NS	NS	NS
Fitness center	0.87 (1.13)	0.63 (0.83)	1.34 (1.16)	NS	NS	NS
Organized sports	0.30 (0.76)	0.27 (0.39)	0.59 (0.99)	NS	NS	NS
Other activities	0.50 (0.68)	0.56 (0.65)	0.49 (0.63)	NS	NS	NS
***Five-year follow-up***						
Walking^a^	2.10 (1.21)	1.81 (1.00)	2.26 (1.63)	NS	NS	NS
Biking	0.78 (1.38)	0.39 (0.78)	1.60 (2.16)	NS	NS	NS
Swimming	0.33 (0.92)	0.08 (0.12)	0.43 (0.70)	NS	NS	NS
Skiing^b^	0.56 (0.92)	0.21 (0.33)	0.49 (0.80)	NS	NS	NS
Fitness center	0.91 (1.30)	0.32 (0.64)	1.19 (1.13)	0.051	**0.002**	0.077
Organized sports	0.38 (1.10)	0.42 (0.80)	0.51 (0.83)	NS	NS	NS
Other activities	0.37 (0.52)	0.61 (0.62)	0.44 (0.40)	NS	NS	NS

Data are presented as self-reported weekly frequency of listed activities. Difference between the groups at each timepoint was tested with Kruskal-Wallis test and if significant tested with Dunn’s test post-hoc. ^a^Walking: as transport, recreational walking, or hiking, ^b^cross-country skiing. Abbreviations: MICT: moderate-intensity continuous training; HIIT: high-intensity interval training; NS: not significant.

### White matter hyperintensities

For the manual WMH volumes, the intra-rater reliability calculated with ICC was 0.99 (95% CI = 0.99 to 1.00) for rater 1 and 0.99 (95% CI = 0.96 to 1.00) for rater 2 indicating excellent agreement. The inter-rater reliability, evaluated in a longitudinal linear mixed model analysis, showed a significant association between WMH and WM-hypointensity volume (estimate = 0.19, 95% CI = 0.15 to 0.22, *p* < 0.001) while no interaction was present between rater and WMH volume (estimate = 0.01, 95% CI = −0.02 to 0.05, *p* = 0.486). Additionally, rater was not associated with WM-hypointensity volume (estimate = −0.19, 95% CI = −0.76 to 0.37, *p* = 0.503). Thus, the inter-rater analysis indicated a similar association between both raters and WMH and WM-hypointensity volumes.

Figure 2 shows the development in WMH volume over time for all participants. The WMH volume varied between participants at baseline (lowest volume: 0, highest volume: 36‰ of ICV) and increased over time. Table 6 shows WMH, PWMH, DWMH and WM-hypointensity volumes at each timepoint during the intervention period. See Tables 7 and 8 for statistical evaluations of time and age effects.

**Table 6:** Total (A) and relative to ICV (B) WMH, PWMH, DWMH and WM-hypointensity volumes at each timepoint for all participants, given as median and interquartile range.

**Time**	**WMH**	**PWMH**	**DWMH**	**WM-hypointensity**
**(A) Total volume (cm^3^)**				
Baseline	4.1 (8.4)	3.3 (6.5)	0.6 (2.3)	3.5 (3.2)
One-year follow-up	5.7 (12.1)	4.7 (8.0)	0.9 (2.7)	4.0 (3.3)
Three-year follow-up	8.8 (14.6)	7.1 (11.3)	1.3 (2.8)	5.0 (5.5)
Five-year follow-up	19.2 (14.2)	15.7 (12.7)	3.0 (3.6)	6.1 (6.0)
**(B) Volume relative to ICV (‰ of ICV)**				
Baseline	2.5 (5.4)	2.2 (3.9)	0.4 (1.4)	2.2 (2.0)
One-year follow-up	3.5 (7.6)	3.1 (5.1)	0.6 (1.6)	2.5 (2.2)
Three-year follow-up	5.6 (8.7)	4.4 (5.9)	0.7 (1.6)	3.0 (3.3)
Five-year follow-up	11.9 (7.8)	10.3 (7.1)	1.8 (2.0)	3.5 (3.4)

In (B) the WMH, PWMH, DWMH and WM-hypointensity volumes were divided by ICV and multiplied by 1000, i.e., reported as ‰ of ICV. Abbreviations: WMH: white matter hyperintensities; PWMH: periventricular WMH; DWMH: deep WMH; WM: white matter; IQR: interquartile range.

**Table 7:** The effect of the five-year intervention on WMH, PWMH, DWMH and WM-hypointensity volumes in the MICT and HIIT groups compared to the control group.

**Predictors**	**WMH**			**PWMH**			**DWMH**			**WM-hypointensity**		
	**Est.**	**CI (95%)**	***p*-value**	**Est.**	**CI (95%)**	***p*-value**	**Est.**	**CI (95%)**	***p*-value**	**Est.**	**CI (95%)**	***p*-value**
Women	1.95	−1.29, 5.19	0.235	0.83	−1.13, 2.79	0.404	1.14	−0.35, 2.62	0.131	−0.80	−1.83, 0.22	0.124
Age	0.28	−0.56, 1.12	0.507	0.48	−0.03, 0.98	0.067	−0.14	−0.51, 0.23	0.447	0.33	0.06, 0.60	**0.016**
1-Year	1.03	−0.71, 2.77	0.245	0.47	−0.69, 1.64	0.424	0.81	0.16, 1.46	**0.015**	−0.03	−0.62, 0.56	0.921
3-Year	2.35	−0.58, 5.27	0.115	1.17	−0.67, 3.01	0.212	1.22	0.01, 2.44	**0.049**	0.09	−0.87, 1.04	0.856
5-Year	7.58	3.00, 12.16	**0.001**	4.50	1.67, 7.33	**0.002**	2.62	0.65, 4.59	**0.009**	0.53	−0.94, 2.00	0.477
MICT	−0.89	−5.19, 3.42	0.683	−0.59	−3.24, 2.05	0.659	−0.24	−2.16, 1.69	0.808	−0.23	−1.61, 1.15	0.739
HIIT	−2.11	−6.02, 1.81	0.288	−1.54	−3.94, 0.86	0.205	−0.30	−2.05, 1.45	0.733	−0.72	−1.97, 0.53	0.258
MICT*1-year	−0.41	−2.66, 1.83	0.718	−0.18	−1.76, 1.41	0.828	−0.31	−0.98, 0.36	0.368	−0.31	−1.12, 0.51	0.457
MICT*3-year	0.77	−1.55, 3.09	0.515	0.61	−1.03, 2.24	0.466	0.05	−0.65, 0.74	0.899	0.12	−0.71, 0.95	0.770
MICT*5-year	0.97	−1.34, 3.28	0.408	1.20	−0.43, 2.83	0.147	0.10	−0.60, 0.79	0.783	−0.57	−1.39, 0.25	0.174
HIIT*1-year	0.42	−1.58, 2.43	0.679	0.20	−1.21, 1.61	0.277	−0.21	−0.81, 0.39	0.498	−0.03	−0.74, 0.69	0.942
HIIT*3-year	0.71	−1.33, 2.76	0.493	0.52	−0.92, 1.95	0.480	−0.11	−0.73, 0.50	0.719	−0.16	−0.89, 0.56	0.658
HIIT*5-year	1.38	−0.71, 3.47	0.193	1.46	−0.00, 2.92	0.050	−0.25	−0.88, 0.38	0.439	−0.37	−1.10, 0.37	0.326
AIC			2095.1			1812.4			1330.1			1379.2

WMH, PWMH, DWMH, WM-Hypointensity volumes were corrected for ICV and multiplied by 1000 (‰ of ICV). All analyses were adjusted for age, sex, and baseline outcome values. The control group was the reference group. There were no interactions between sex, age, time (year one, three and five) or the training interventions (MICT and HIIT) and these terms were therefore removed from the statistical models. Abbreviations: WMH: white matter hyperintensities; PWMH: periventricular WMH; DWMH: deep WMH; WM: white matter; Est.: estimates; CI: confidence interval; MICT: moderate-intensity continuous training; HIIT: high-intensity interval training; AIC: Akaike’s information criterion.

**Table 8:** A longitudinal linear mixed model of WMH, PWMH, DWMH and WM-hypointensity volumes with VO_2peak_ at time of each follow up as a predictor across all participants.

**Predictors**	**WMH**			**PWMH**			**DWMH**			**WM-hypointensity**		
	**Est.**	**CI (95%)**	***p*-value**	**Est.**	**CI (95%)**	***p*-value**	**Est.**	**CI (95%)**	***p*-value**	**Est.**	**CI (95%)**	***p*-value**
Women	2.21	−1.04, 5.45	0.181	0.98	−0.99, 2.96	0.327	1.23	−0.32, 2.78	0.118	−0.93	−1.96, 0.10	0.076
Age	0.27	−0.56, 1.10	0.518	0.47	−0.04, 0.97	0.071	−0.18	−0.57, 0.21	0.369	0.30	0.04, 0.57	**0.025**
Year one	1.03	−0.47, 2.52	0.176	0.49	−0.47, 1.45	0.313	0.77	0.10, 1.44	**0.024**	−0.03	−0.53, 0.47	0.919
Year three	2.72	−0.04, 5.48	0.053	1.49	−0.23, 3.20	0.088	1.37	0.08, 2.65	**0.038**	0.23	−0.67, 1.12	0.617
Year five	8.39	3.96, 12.81	**<0.001**	5.40	2.68, 8.11	**<0.001**	3.05	0.97, 5.13	**0.004**	0.47	−0.94, 1.89	0.509
VO_2peak_	−0.00	−0.12, 0.12	0.998	−0.01	−0.09, 0.07	0.778	−0.00	−0.05, 0.05	0.900	−0.03	−0.07, 0.01	0.153
AIC			1986.4			1713.3			1422.7			1314.5

WMH, PWMH, DWMH, WM-hypointensity volumes were corrected for ICV and multiplied by 1000 (‰ of ICV). All analyses were adjusted for age, sex, and baseline outcome values. The control group was the reference group. There were no interactions between sex, age, time (year one, three and five) or VO_2peak_ and these terms were therefore removed from the statistical models. Abbreviations: WMH: white matter hyperintensities; PWMH: periventricular WMH; DWMH: deep WMH; WM: white matter; Est.: estimates; CI: confidence interval; VO_2peak_: peak oxygen uptake; AIC: Akaike’s information criterion.

### Intervention results

The linear mixed model investigating the effect of MICT and HIIT compared to the control condition on WMH volume during the five-year RCT did not reveal an interaction between group and time on WMH volume (Table 7). Only time at five years was significantly positively associated with WMH volume. Similar results were uncovered for PWMH volume. However, DWMH volume was significantly positively associated with time at year one, three and five. WM-hypointensity volume was not associated with group, group*time or time, but there was a significant effect of age.

The analysis assessing the difference between the combined supervised exercise group (MICT&HIIT) versus control found a significant interaction between group and time at year five for the PWMH volume (Supplementary Table 1), with participants in the supervised exercise group having larger PWMH volume with time (Supplementary Figure 1). The results were similar in the supplemental analyses, which included WMH risk factors: current smoking, hypertension, BMI and HADS score as predictive variables in the same models as above (results not shown).

Across the three groups, no significant associations between VO_2peak_ and WMH volume were found at any of the follow-ups (Table 8). Similar results were present for PWMH, DWMH and WM-hypointensity volumes. The results did not change when including current smoking, hypertension, BMI and HADS score as predictive variables in the supplemental analyses with WMH, PWMH, DWMH or WM-hypointensity volume as outcomes (results not shown).

Across all participants, the ‘model of changes’ demonstrated no effect of change in VO_2peak_ on change in WMH, PWMH, DWMH, or WM-hypointensity volume (Table 9). There was a significant effect of age; the older a participant was, the greater the increase in WMH and PWMH volume. Being a woman was associated with a greater increase in DWMH volume.

**Table 9:** Linear mixed ‘model of changes’ analyses with yearly change in VO_2peak_ (ΔVO_2peak_) as predictor of yearly change in WMH, PWMH, DWMH, and WM-hypointensity volumes across all participants.

**Predictors**	**WMH change**			**PWMH change**			**DWMH change**			**WM-hypointensity change**		
	**Est.**	**CI (95%)**	***p*-value**	**Est.**	**CI (95%)**	***p*-value**	**Est.**	**CI (95%)**	***p*-value**	**Est.**	**CI (95%)**	***p*-value**
Women	0.28	−0.34, 0.90	0.380	0.17	−0.23, 0.58	0.400	0.26	0.03, 0.50	**0.029**	−0.09	−0.29, 0.11	0.363
Age^a^	0.18	0.04, 0.32	**0.014**	0.16	0.07, 0.26	**0.001**	−0.02	−0.07, 0.03	0.396	0.03	−0.01, 0.08	0.136
ΔVO_2peak_	0.09	−0.01, 0.19	0.088	0.04	−0.03, 0.11	0.266	0.02	−0.02, 0.05	0.291	0.01	−0.02, 0.04	0.484
AIC			985.2			800.4			531.0			476.6

WMH, PWMH, DWMH, WM-hypointensity volumes were corrected for ICV and multiplied by 1000 (‰ of ICV). Yearly changes were calculated from inclusion to one-year, one-year to three-year, and three-year to five-year of the intervention. There were no interactions between sex, age, or VO_2peak_ and these terms were therefore removed from the statistical models. ^a^The participants’ mean age at each time interval. ^2^Yearly change in peak oxygen uptake of the three follow up intervals. Abbreviations: WMH: white matter hyperintensities; PWMH: periventricular WMH; DWMH: deep WMH; WM: white matter; Est.: estimates; CI: confidence interval; VO_2peak_: peak oxygen uptake.

Baseline VO_2peak_ did not predict WMH change over five years (*p* = 0.754).

## DISCUSSION

In this MRI sub-study of participants from the RCT Generation 100 Study, we did not find evidence of five years of supervised MICT or HIIT intervention attenuating the development of WMH compared to the control group following the national physical activity guidelines. Neither did we uncover a positive association between cardiorespiratory fitness (VO_2peak_) and WMH, nor change in VO_2peak_ on change in WMH volume at any timepoint during the intervention. Likewise, no intervention or VO_2peak_ effect on PWMH, DWMH and WM-hypointensity volumes were present. Including known risk factors for WMH as predictors in the statistical models did not alter these results. The lack of a group difference in WMH volume was present even though all three groups adhered well to their respective training regimes, with the HIIT group training at a higher intensity and partaking in different types of activities than the MICT and control groups. However, in the combined supervised exercise group, a significant interaction between time and group was uncovered, with the intervention group experiencing greater PWMH volume growth after five years. Taken together, supervised exercise was not found to be beneficial above following national physical activity guidelines, and level of cardiorespiratory fitness was not found to attenuate the growth of the investigated WMH measures contrary to our predictions.

Our results were in line with the recent two-year AIBL active intervention study with subject-specific self-administered physical activity of medium intensity versus usual care/lifestyle advice in older community-dwelling adults of similar age as in our study [24]. In that study, no positive effect of the intervention was uncovered for manually derived longitudinal WMH volume from 3D FLAIR scans acquired at 3T [24]. A possible explanation for the lack of a group effect in both the AIBL active and our study could be similarities in fitness levels between the intervention and control groups. In Venkatraman et al. [24], fitness/motor test scores were similar in both groups, while in our study, the groups had similar VO_2peak_ during the intervention. No effect on WMH development has also been reported in the PROMOTE study with thrice weekly aerobic exercise versus usual care in adults aged 55 years and older with MCI [22] and in the multidomain intervention versus usual care, FINGER study, in an at-risk population [23]. Since physical activity was only one of several approaches implemented in the FINGER study, it is not directly comparable to our study. Still, the FINGER study reported a positive effect of their multidomain intervention on processing speed in the participants with the largest structural brain reserve [29]. In our study cohort, processing speed was positively associated with VO_2peak_ across all participants, and increasing VO_2peak_ during the intervention improved working memory [30]. Taken together, this might suggest that interventions including exercise and/or physical activity provide brain functional benefits even if brain structural benefits cannot be detected.

In contrast to the intervention studies, which did not report intervention effects on WMH, two three-year observational studies on the effect of physical activity on WMH volumes showed a positive association between level of physical activity on WMH volume over time, but only when comparing the most and the least physically active, cognitively unimpaired participants [26, 31]. In these studies, the most physically active groups with significantly lower WMH volume at the end of the intervention exercised at >1200 metabolic equivalent of task (MET)-minutes/week and >1875 kcal per week, respectively. Most participants in our study did not exercise at this level. Moreover, the reference groups in the aforementioned studies spent <600 MET-min/week and <217 kcal and on physical activity, respectively. This is lower than the physical activity levels of our control group. This could imply that very vigorous and frequent physical activity is required to attenuate WMH volume over time.

In the combined exercise group, a significant group*time effect was uncovered at five years, but contrary to our prediction, PWMH growth was greatest in the intervention group. Figure 2 depicting overall WMH volume over time suggests a greater WMH growth in MICT and HIIT compared to controls over time, but a clear change in trajectory was only observed in the combined MICT&HIIT group (Supplementary Figure 1). The periventricular region is supplied by short, penetrating high flow vessels sensitive to hypertension and is linked to stroke risk [9, 32, 33]. Furthermore, PWMH volumes correlate with several genes connected to vascular function and vascular diseases in the brain and heart [8]. Since exercise improves vascular function and prevents cardiovascular disease [34–36], a specific adverse effect of the intervention on PWMH volume over time was unexpected. However, we have previously shown a time*group interaction for hippocampal volume in the same cohort with the HIIT and combined HIIT and MICT groups experiencing faster hippocampal atrophy rate from year three [37]. Such negative effects on brain structure could relate to the older brain being more sensitive or more likely to experience hypoperfusion during intense exercising [25].

The similar VO_2peak_ level in the three groups across the intervention period was unexpected given that the groups exercised according to their assigned regime throughout the five-year period. HIIT is anticipated to increase VO_2peak_ the most [38], and a small but significantly higher VO_2peak_ was present in HIIT compared to MICT and control groups in the full RCT Generation 100 Study sample [27]. In the MRI sub-study, the participants had a significantly higher VO_2peak_ at baseline than the rest of the RCT Generation 100 participants, which might have contributed to the lack of a group difference, even if the HIIT group in the MRI sample consistently exercised at a higher intensity level that the MICT and control groups. Furthermore, VO_2peak_ at baseline did not predict change in WMH volume over five years. This was at odds with findings for cortical volumes in the same cohort, where VO_2peak_ at baseline was positively associated with cortical volume at five years [37]. Different brain tissues may hence be differentially sensitive to the effect of exercising and cardiorespiratory fitness levels. Since the mean VO_2peak_ level in our sample was similar to that in people of the same age in a large Norwegian general population study [39], our results should be generalizable to older cognitively intact community-dwelling adults. The absence of an association between VO_2peak_ and WMH volume and change in VO_2peak_ and change in WMH volume at any timepoint during the intervention makes it unlikely that VO_2peak_ by itself is a central mechanism in preventing WMH. Since non-exercise/physical activity intervention studies in adults between 40–90 years of age find significantly reduced growth of WMH following pharmacological (e.g., antihypertensives, intranasal insulin), life-style (e.g., diabetes mellitus type 2), and physiological (e.g., preconditioning) interventions [40–43], it might be that aerobic exercise intervention alone is less effective at reducing WMH compared to targeting other or several mechanisms associated with WMH.

The WMH and WM-hypointensity volumes in the participants at baseline were highly similar to findings of older adults within the same age range using the same type of scans and WMH delineation methods [44, 45], as well as in published longitudinal studies on physical activity and exercise [24, 26]. Moreover, the Generation 100 participants had the expected increase in WMH volume with time. However, the rate of WMH volume growth in our sample was in the higher end of previously published data [46–48]. It is to be mentioned that both cross-sectional volume and longitudinal WMH volumes vary substantially between publications, probably due to the many different methodologies used for WMH measurements [26, 47–50]. In our study, only DWMH volume increased at each follow-up, while total WMH and PWMH volumes were significantly increased only at five years. The faster growth of DWMH could be due to the greater area available for growth. Presence and growth of WMH are associated with health factors such as hypertension, weight, and mental health, which affect white matter long before WMH become visible [51, 52]. Many of these factors interact with physical activity over time, representing time-dependent confounders, which makes it difficult to unravel the relationships between the different mechanisms involved in WMH formation [53]. After 70 years of age, it might be that changes in white matter related to lifelong exposure to physiological, health and environmental factors have come too far to be altered by an exercise intervention. Other effects of physical activity and aerobic exercising than improved VO_2_, such as better cardiovascular health, body weight control, and exercise-induced increases in levels of substances in blood (e.g., BDNF, lactate), have been suggested as important, beneficial mechanisms of exercise on the brain [54]. Since there were no differences in the demographic and clinical characteristics between the three groups in our study at any timepoint during the intervention, and adding WMH risk factors to the models did not alter the results (supplemental analyses), it seems unlikely that differences in clinical health related to MICT, HIIT or physical activity according to national guidelines could have affected our results.

Women had a greater DWMH volume increase across the five-year intervention compared to men in our study. Previous studies have reported sex differences in PWMH volume [13] and total WMH volume, but with a low explanatory power [55]. Our results imply that growth in WMH volume is more related to sex than cross-sectional WMH volume per se in the 70+ age group. Given that DWMH are a risk factor of dementia [12] which women are at an increased risk of [56], this finding has potential clinical relevance.

WM-hypointensity volume was, like WMH volume, not associated with group or group*time interaction. The WM-hypointensity measure shares characteristics with automated segmentation algorithms for WMH obtained from T2-weighted/FLAIR scans in that both report smaller WMH volumes mainly located in deep white matter and are limited to voxels with markedly higher (T2-weighted) or lower (T1-weighted) signal intensity, considered to reflect more severely affected white matter. Our results suggested that supervised HIIT and MICT exercising were not effective in preventing the growth of this type of age-related WM change either. Most observation and intervention studies examining relationships between physical activity and WMH have used (semi-)automated WMH segmentation methods, and most of these report positive results [26, 49, 57–59]. We were not able to find a similar positive relationship with WM-hypointensity volume. The consistent findings with manually and automatically derived WMH volumes on this study demonstrated that differences in WMH segmentation methods cannot explain our null results.

### Strengths and limitations

The strengths of this study included the general population-based sample, the limited age range, even distribution between men and women, prospective, RCT design, long intervention period, clinical measures, ergospirometry VO_2_ assessments, detailed physical activity data and brain MRI at baseline, one-, three- and five years. MRI was obtained on the same scanner with the same coil and the same scan protocol at all timepoints. The WMH volumes were obtained by manual delineation, considered the gold standard of WMH quantification [44, 60], as well as with an automated method, and these methods were significantly associated. The scans for WMH delineation were 3D FLAIR scans, which are considered to allow for the highest reliability and reproducibility of WMH volume measurements, and deemed the most sensitive method to uncover a change in WMH volume over time [61–63]. The two raters performed highly consistent and similar measurements. All data were missing at random. Correction for baseline values was implemented as recommended by Twisk [64]. The sample size was determined based on publications available at time of the application to the ethical committee and should be able to uncover group differences. Nevertheless, only the analysis with the combined supervised exercise group revealed a significant time*group interaction, although not in the expected direction. An increase in power by combining the MICT and HIIT groups (higher n and two groups) was likely the reason for this since the estimates from the mixed linear model analyses for PWMH volumes in the MICT and HIIT versus the control group (Table 7) and the combined MICT&HIIT group versus the control group (Supplementary Table 1) were quite similar. We added the table with WMH volumes at each timepoint to guide future power analysis in this research area, as the cross-sectional and longitudinal studies published since this study started report varying results [19, 20, 22, 23, 26, 40, 47–50, 58, 59, 65–68].

Our participants were on average quite healthy with fewer current smokers and less diabetes and hypertension than in average 67–79 years old Norwegians [69, 70]. The participants in the MRI study of the Generation 100 study were also more educated, had a higher mean VO_2peak_ level and lower blood fat level than those not volunteering for the MRI sub-study. Inclusion of a control group might have precluded uncovering an effect of the supervised exercise intervention, but it was considered unethical not to have the control group follow the national guidelines on physical activity based on the current evidence regarding somatic health and mortality. Many previous studies examining WMH and physical activity/exercise have included at-risk populations or hospital samples [22, 23, 26, 31, 40, 71]. Since WMH is common in older adults [1], it is also important to investigate this phenomenon in the general population, including healthy older adults, to devise inclusive strategies for optimal brain aging.

## CONCLUSION

This is the first five-year intervention study implementing exercise interventions at two intensities and a control group, recruited from the general population of community-dwelling older adults born between 1936 and 1942. The exercise intervention did not influence WMH, PWMH, DWMH, or WM-hypointensity volume growth compared to the control group following national recommendations for physical activity. Neither was VO_2peak_ at any timepoint nor change in VO_2peak_ associated with any WMH measurement in any group, and baseline VO_2peak_ did not predict WMH growth. Exercise in old age has several benefits and should be recommended to improve overall health but taking part in MICT or HIIT does not protect against WMH progression compared to following national physical activity guidelines.

## MATERIALS AND METHODS

### Ethics

The project was approved by the Regional Committee for Medical Research Ethics, Central Norway (2012/849) and adhered to the Declaration of Helsinki. All participants signed an informed written consent before inclusion.

### Study population

The participants were from the RCT Generation 100 Study (NCT01666340, http://clinicaltrials.gov/ct2/show/NCT01666340) approved separately by the Regional Committee for Medical Research Ethics, Central Norway (2012/381 B) [28]. The RCT assessed the effect of five years of twice weekly supervised MICT or HIIT compared to the national recommendations of at least 30 minutes moderate-intensity physical activity almost every day [72] on all-cause mortality in older adults [27].

In 2012, invitation letters were sent to 6 966 adults (3 721 women) born between 1936–1942 and registered in the Norwegian National Population Registry with a permanent home address in Trondheim municipality. Of these, 1 790 showed an interest, and 1 567 (790 women) passed the inclusion criteria while 223 were excluded. Exclusion criteria were any condition or disease precluding partaking in an exercise intervention and diagnosed dementia as well as participation in other exercise intervention studies [28]. The participants were informed of the possibility of also taking part in a neuroimaging investigation during the baseline data collection in the Generation 100 RCT. Exclusion criteria for the MRI study were limited to standard MRI contraindications (e.g., implanted electronic medical devices) and brain pathology, which would interfere with image analysis.

After inclusion, the Unit for Applied Clinical Research, NTNU, used a web-based approach to randomize the participants 2:1:1, stratified by sex and cohabitation status (living with someone versus alone) into following the national physical activity guidelines (i.e., >30 minutes of moderate-intensity physical activity almost every day) (control group, *n* = 780), or supervised exercise with either MICT (*n* = 387) or HIIT (*n* = 400) [28]. The supervised MICT sessions consisted of 50 minutes of continuous workout or exercise at about 70% of peak heart rate corresponding to a rating of perceived exertion of approximately 13 on the Borg scale [73]. The supervised HIIT sessions included 10-minute warm-up followed by 4 × 4 minute intervals between 85–95% peak heart rate corresponding to a rating of perceived exertion of approximately 16 on the Borg scale. Between the intervals, there were three-minute active breaks. Participants could perform their training sessions individually at their assigned intensity level after instruction. Every sixth week, the MICT and HIIT groups attended mandatory spinning classes where they exercised with a heart rate monitor to ascertain compliance with the prescribed training intensity. Of those included in the Generation 100 Study, 108 participants were interested in undergoing neuroimaging. Of these, two were excluded due to MRI contraindications, and one was excluded due to pre-existing brain pathology leaving 105 participants to be included.

Sample size for the neuroimaging project was calculated at the time of the application to the ethical committee based on previously WMH volume growth which varied greatly [50, 74], giving group sizes ranging from 4 to 52 individuals to uncover significant group differences.

Baseline MRI acquisition started in August 2012 and lasted till June 2013. Follow-ups were performed one, three, and five years after baseline data collection, with five-year MRI scans collected between August 2017 and June 2018.

For the MICT and HIIT groups, adherence to the prescribed intervention was based on reported frequency, duration, and intensity of exercise. This information was obtained from the physical activity questionnaires at the one-, three- and five-year follow-ups [28]. As per the RCT protocol, adherence to the assigned program was met if the participant fulfilled at least 50% of the prescribed sessions [28]. For HIIT, adherence was defined as exercising at least ≥30 minutes ≥15 on the Borg scale per week, and for MICT at least ≥30 minutes at 11–14 on the Borg scale per week. For the controls, adherence was based on performing at least ≥75 minutes of physical activity (i.e., including all intensities, Borg 6–20) per week. Adherence for each group was calculated as the number of participants adhering to the prescribed exercise/physical activity divided by the total number of participants in the group at that timepoint and presented as a percentage. From the same questionnaire, exercise frequency per week, exercise duration in minutes per session, and intensity rated with the Borg scale were derived for each participant at the follow ups. Finally, the frequency of performing different types of activities was assessed from the following questions: “How often do you do the following: 1. Walk: a) as a way of transportation, b) recreational walking, c) hiking in nature); 2. Bike; 3. Swim; 4. Ski; 5. Train at a fitness center; 6. Participate in organized sports; 7. Participate in other physical activities”. The response options were: “Never” scored as 0; “Rarely” scored as 0.25; “1–3 times a month” scored as 0.5; “once a week” scored as 1, “2–3 times a week” scored as 2.5; “4–6 times a week” scored as 5; and “Daily” scored as 7. The weekly frequencies of the different activities were used to compare the different groups.

### Demographic, clinical data and cardiorespiratory fitness measurement

All participants completed a questionnaire concerning their demographical information and health. Education was stratified into primary school, high school/vocational school, and higher education. Smoking was registered as ‘current smoker’ (yes/no) at all timepoints and as pack-years at baseline, i.e., the number of packs of cigarettes smoked per day multiplied by the number of years the person smoked.

Clinical measurements were obtained at baseline and one, three, and five years after inclusion. Height, weight, waist circumference, and blood pressure (BP), were measured using best clinical practices [28]. Fasting blood was drawn and analyzed for serum triglycerides (TG), glucose, low-density lipoprotein (LDL), high-density lipoprotein (HDL), total cholesterol, glycosylated hemoglobin (HbA1c), and high-sensitive CRP (hsCRP). A diagnosis of diabetes (type 1 and type 2) was based on reporting a diagnosis of diabetes, and/or fasting blood glucose ≥7.0 mmol/L, and/or HbA1c ≥48 mmol/mol [75]. Hypertension was defined as reporting a hypertension diagnosis, and/or use of antihypertensives, and/or systolic BP ≥140 mmHg and/or diastolic BP ≥90 mmHg [76]. The validated Norwegian version of the Hospital Anxiety and Depression Scale (HADS) was used to assess anxiety and depression levels, with higher scores indicating increased symptom burden (range 0–42) [77, 78]. At the 5-year assessment, the Norwegian validated Montreal Cognitive Assessment (MoCA) tool was used to evaluate cognition [79]. The score range is 0 to 30, where a lower score denotes lower cognitive ability. The raw scores are presented and the cut offs for a diagnosis of mild cognitive impairment (MCI) is a score of 21 for primary, 22 for secondary, and 24 for high educational attainment for the age group 75–85 years based on Scandinavian norms [80].

Cardiorespiratory fitness was assessed as VO_2_ (mL·kg^−1^·min^−1^) obtained with graded maximal exercise testing on a treadmill or an ergometer bicycle [81]. Participants with previous heart diseases were tested under ECG monitoring, and participants with known cardiovascular disease were tested according to the American College of Cardiology/American Heart Association guidelines for exercise testing of patients with known cardiovascular disease [82]. VO_2max_ is reached when the respiratory exchange ratio is ≥1.05 and VO_2_ does not increase more than 2 mL between two 30-second periods despite increased workload. If the participant was unable to meet the VO_2max_ criterion, VO_2peak_ was estimated instead. VO_2peak_ was calculated from the mean of the three highest VO_2_ measurements across a consecutive 10 second period. For participants with VO_2max_ this value was used while VO_2peak_ was used for those who could not reach maximal oxygen uptake in this study. The term peak oxygen uptake (VO_2peak_) refers to the combination of these measures in this study.

### Magnetic resonance imaging

#### MRI acquisition

Brain MR imaging was acquired on the same 3T Siemens Skyra scanner equipped with a 32-channel head coil. A standardized Generation 100 Study MRI protocol was used at all timepoints. In this study, the 3D T1-weighted, T2-weighted, and T2-weighted FLAIR scans were used (Table 10). The mean intervals (SD) between the MRI scans at baseline, one-, three-, and five-year follow-up were 475 (13) days, 628 (21) days, and 773 (33) days, respectively.

**Table 10:** MRI scan parameters for scans acquired at 3T used in this study.

**Parameter**	**3D T1**	**3D T2**	**3D T2 FLAIR**
**Orientation**	Sagittal	Sagittal	Sagittal
**TR**	1900 ms	3200 ms	5000 ms
**TE**	3.16 ms	412 ms	388 ms
**TI**	900 ms	n/a	1650 ms
**No. of Slices**	192	176	176
**Slice Thickness**	1.0 mm	1.0 mm	1.0 mm
**FOV**	256 × 256 mm	250 × 250 mm	256 × 256 mm
**Matrix (resolution)**	256 × 256	256 × 256	256 × 256
**Flip Angle**	9°	T2 variable	T2 variable
**Turbo Factor (ETL)**	224	282	278
**Averages**	1	1	1

Abbreviations: FLAIR: fluid-attenuated inversion recovery; TR: repetition time; TE: echo time; TI: inversion time; FOV: field-of-view; ETL: echo train length.

#### Manual and automated processing of brain MRI scans

**Figure 3:**
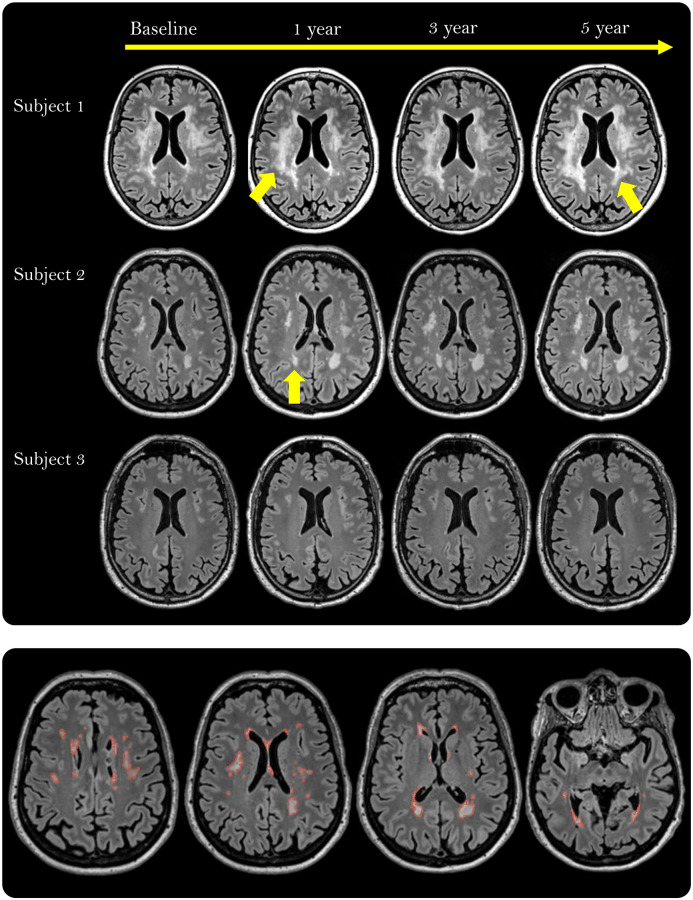
**White matter hyperintensities (WMHs) depicted as white, well-defined lesions or confluent areas in white matter on fluid-attenuated inversion recovery (FLAIR) scans in three participants during the 5-year intervention period.** The yellow arrows point to areas with growing WMH lesions (Subject 1) or a new lesion (Subject 2). The lower row shows the manually delineated WMHs (red outline) across 4 out of 176 slices in one participant.

All image analysis was performed blinded for the participants´ group adherence, demographic and clinical information. Manual segmentation of WMH was performed by delineating the hyperintense regions in WM on the FLAIR scans from the four different timepoints using the software Multi-image Analysis GUI (Mango) ver. 4.0.1 (http://rii.uthscsa.edu/mango/) and a Wacom Intuos pen tablet model CTL-480 (Wacom Co., Ltd., Saitama, Japan) (Figure 3). One rater performed the manual delineation at baseline and one-year follow-up and another rater at three- and five-year follow-up. To make segmentations comparable, rater 1 instructed rater 2, and WMH delineation in the first 25 scans from the three-year follow up was performed side-by-side with already delineated WMH in the same brains at the one-year follow up. Each rater delineated WMH again in a random selection of ten participants, blinded for previous results by the same rater for intra-rater reliability analysis.

To investigate if certain regions of WMH were more sensitive to the effects of the intervention and/or VO_2peak_, WMH were divided into PWMH and DWMH. Likewise, associations between intervention group and/or VO_2peak_, and automatically derived WM-hypointensity volume from T1-weighted scans, which are considered to reflect more severely affected WMH regions [44, 83, 84], were assessed.

**Figure 4:**
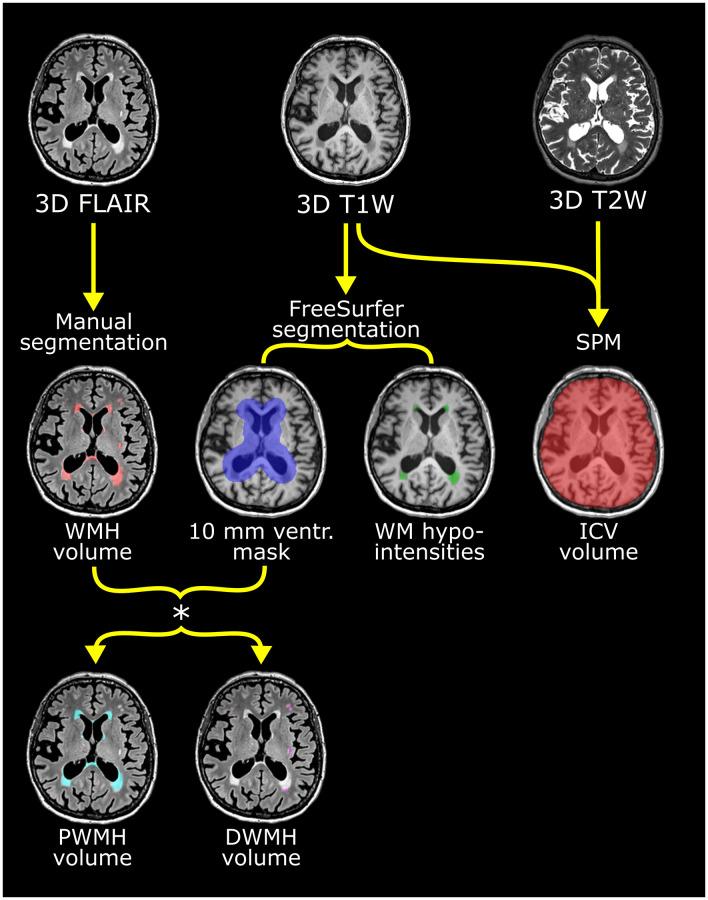
**Flow chart of the multiparametric analysis of the brain MRI scans exemplified with scans from one participant.** Upper row shows the 3D FLAIR, T1- and T2-weighted scans used. See Table 10 for scan details. Manual segmentation of white mater hyperintensities (WMH) was performed on the FLAIR images. From the T1-weighted scan, the ventricular (ventr.) mask derived with FreeSurfer (shown in violet in middle row) was used to stratify WMH into periventricular white mater hyperintensities (PWMH) (located <10 mm from the ventricular edge) and deep white mater hyperintensities (DWMH) (≥10 mm from ventricular edge) as seen in the lower row. PWMH are depicted in cyan and DWMH in white. White matter (WM)-hypointensity volume was also derived from the FreeSurfer analysis (middle row) and shown as green lesions. Intracranial volume (ICV) was calculated from the T1- and T2-weighted scans using SPM. These segmentations were performed separately for the scans from baseline, one-, three- and five years.

The flow of the image analysis pipeline after manual segmentation is depicted in Figure 4. From the manually assessed WMH mask, the total WMH volume and the volumes of PWMH and DWMH were obtained. For each participant, the PWMH and DWMH volumes were acquired at each timepoint by applying the lateral ventricular mask from the FreeSurfer analysis (v 6.0.0-2) of the 3D T1-weighted scans (http://surfer.nmr.mgh.harvard.edu/) [85] (Figure 4). The ventricular mask was rigid-body aligned from the 3D T1-weighted to the FLAIR scan in ANTS (v 2.3.1). Subsequently, the ventricular mask was morphologically dilated by 10 mm using the ImageMath program in ANTS (Figure 4). PWMH were WMH located within 10 mm from the ventricular edge, while DWMH were WMH located beyond 10 mm from the ventricular edge. The 10 mm cut off was chosen as it is widely used and provides a separation between PWMH and DWMH with regard to differences in the two regions’ associations with cardiovascular risk factors, physiological parameters and cognitive scores [13, 86, 87]. The fit of the ventricular mask was visually quality assessed for all timepoints in all individuals.

The total volume of white matter (WM)-hypointensity, reflecting white matter signal loss on the 3D T1-weighted scans, was obtained by automated segmentation for subcortical structures in FreeSurfer (v 6.0.0-2) (Figure 4).

Intracranial volume (ICV) was estimated in SPM8 (http://fil.ion.ucl.ac.uk/spm) with the automatic reverse brain mask method using the 3D T1- and T2-weighted scans [88] (Figure 4).

The WMH, PWMH, DWMH and WM-hypointensity volumes were normalized by dividing by ICV and multiplied by 1000 (i.e., ‰ of ICV) to make the results more legible. The different WMH measurements were leftward skewed, also after scaling to ICV. The ICV corrected WMH measures were used in the statistical analysis since regression models do not require the dependent variables to be normally distributed.

### Statistical analysis

#### Demographic and clinical data

Analyses of missing data were performed using Little’s test of missing completely at random [89]. The test was performed for all variables.

Demographic and health characteristics at baseline are presented as mean (standard deviation) or median (interquartile range) based on the distribution of the variable. Group comparisons were performed with independent samples *t*-test, Mann-Whitney *U*-test, one-way ANOVA or Kruskal-Wallis test (with Dunn’s test for post-hoc analyses) for continuous and ordinal variables, and Chi-Square-test or Fisher’s exact test for categorical variables.

The development of VO_2peak_ for all participants was assessed using a linear mixed model with VO_2peak_ as the dependent variable, time and group and their interaction as fixed effects, with participant as a random effect, adjusting for sex, age, and the dependent variable (e.g., VO_2peak_) at baseline as recommended by Twisk et al. [90] and Coffman et al. [91]. In this model, the coefficients for the interaction terms give the estimated intervention effects at year one, three, and five. After analysis, the normality of the residuals was checked by visual inspection of QQ-plots. Three residuals deviated from the normal distribution and were considered outliers. A sensitivity analysis excluding these observations gave similar results (data not shown).

#### Intra- and inter-rater reliability

For the ten manually delineated WMH, which were rated twice by the same rater, intra-rater reliability was assessed for each rater using intraclass correlation coefficient (ICC). ICC estimates and their 95% confidence intervals were calculated based on single measurement, absolute-agreement, 2-way mixed-effects model. Interpretation of the ICC for intra-rater reliability was based on values from Portney and Watkins, where ICC values between 0.75 and 0.9 indicate good reliability, and values greater than 0.90 indicate excellent reliability [92].

The known correlation between WMH and WM-hypointensity was used for assessment of the inter-rater reliability [83, 84]. Inter-rater reliability was evaluated in a longitudinal linear mixed model analysis with total WM-hypointensity volume as outcome, and WMH volume at baseline, and after one-, three-, and five years of intervention, with rater, and interaction between rater and WMH volume included in the model. For the inter-rater assessment, the presence of a significant association between WMH and WM-hypointensity volume combined with no effect of rater on WM-hypointensity volume nor interaction between rater and WMH volume, was interpreted as a good correlation between raters and lack of rater-specific effects.

#### Intervention assessment; group effect

We first investigated the effect of MICT and HIIT interventions compared to controls on WMH volume during the five-year RCT. We used a linear mixed model similar to the one previously described, but with total WMH volume normalized to ICV, replacing VO_2peak_ as the dependent variable. Similar linear mixed model analyses were subsequently performed with ICV normalized PWMH-, DWMH- and WM-hypointensity volume as dependent variables to assess if the intervention affected the two WMH regions and/or WM-hypointensity differently. The interaction between group and time was considered the main outcome and included in all analyses. Interactions between group, sex and age were also investigated based on findings in previous literature, but only retained in the model if significant. The normality of residuals for each linear mixed model performed was checked by visual inspection of QQ-plots. If a deviation from normality was observed, the analysis was repeated after removing observations that represented outliers. The analysis without outliers was performed for WMH, PWMH, DWMH and WM-hypointensity volumes. After removing outliers, analyses for WMH, PWMH and WM-hypointensity volume analyses gave similar results (data not shown), and hence only results from the main model were reported. For DWMH, three residuals were confirmed as outliers and the corresponding observations were excluded in the final model which was reported.

In accordance with the main RCT analysis [27], an additional analysis using the aforementioned linear mixed model was applied with a combined supervised exercise group (MICT&HIIT versus controls) as fixed effect instead of including the MICT and HIIT groups separately.

#### Intervention assessment; VO_2peak_ effects

To evaluate the effect of VO_2peak_ on WMH volume across the intervention period, VO_2peak_ at each timepoint was added to the previously described linear mixed model with WMH as dependent variable. Group and the interaction between group and time were excluded as no effect of group on VO_2peak_ was uncovered. The analysis was repeated with PWMH, DWMH and WM-hypointensity as dependent variables. To correct for baseline VO_2peak_, each participant’s four VO_2peak_ values was subtracted by the baseline-value (e.g., one-year follow-up minus baseline value, three-year follow-up minus baseline value, etc.). Correcting for baseline VO_2peak_ did not alter the results and was therefore removed from the final model. All analyses were tested for interactions between VO_2peak_, age, and sex. There were no significant interactions, and they were therefore not included in the final model.

To evaluate the effect of changing VO_2peak_ on changing WMH volume across the intervention, we used the ‘model of changes’ described by Twisk as a more sensitive method for uncovering change over time [64]. Subjects were included as a random effect, and age and sex as the fixed effects. Yearly change in WMH and VO_2peak_ was calculated by subtracting the former year’s value from the following one (e.g., one-year follow-up minus baseline value, etc.). As there were two years between the two last follow-ups, the delta was therefore divided by two to get the ‘yearly’ change in values. The analysis was repeated with PWMH, DWMH and WM-hypointensity volumes as dependent variables. All the models were tested for interactions between VO_2peak_, age and sex. There were no significant interactions, and the final model was applied without interactions.

Finally, we examined if baseline VO_2peak_ were correlated with overall WMH change over five years (five-year WMH volume minus baseline WMH volume) using a linear regression model with five-year change in WMH volume as dependent variable and baseline VO_2peak_ as covariate, adjusted for age and sex.

#### Supplemental analysis including risk factors for WMH

Other factors related to WMH and physical activity/fitness, such as current smoking, hypertension, BMI and HADS score were added to the linear mixed models assessing effects of group and VO_2peak_ on WMH, PWMH, DWMH and WM-hypointensity volume, as some studies report these factors to affect the relationship between WMH and physical activity/fitness measures [17, 18].

The threshold for statistical significance was set to *p* < 0.05. Correction for multiple hypotheses was not done since this is the first five-year exercise intervention study implementing supervised MICT and HIIT interventions and avoiding type 2 errors is important in this setting. The analyses were carried out in SPSS version 27.

## Supplementary Materials

Supplementary Figure 1

Supplementary Table 1
