# Supplementary material for: Five years of exercise intervention at different intensities and development of white matter hyperintensities in community dwelling older adults, a Generation 100 sub-study
Source: Aging (Albany NY). 2022 Jan 18;14(2):596–622. doi: 10.18632/aging.203843 (PMC8833118; doi:10.18632/aging.203843)
Supplement: Supplementary Figure 1 [file aging-14-203843-s001.pdf]

SUPPLEMENTARY FIGURE

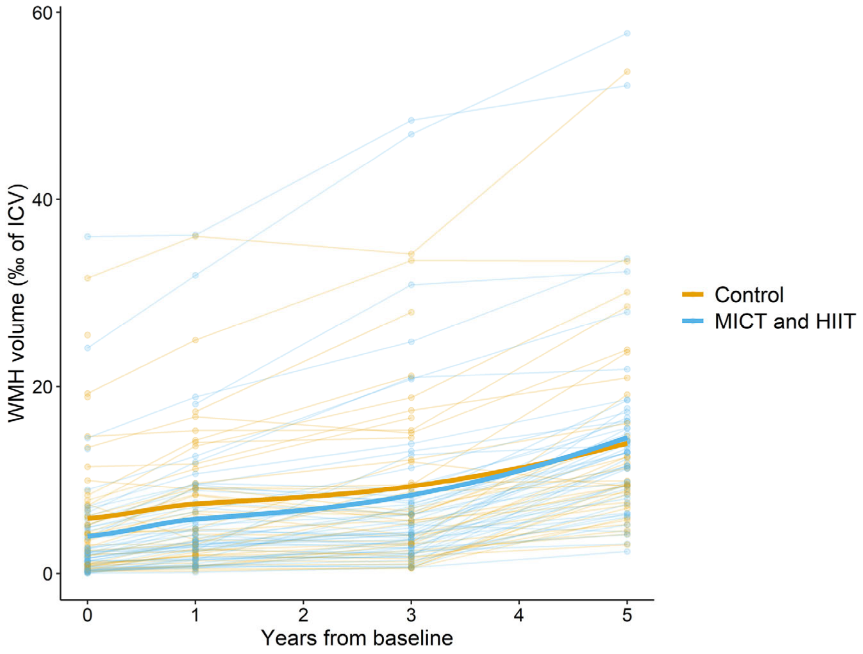

Supplementary Figure 1. Illustration of the interaction effect demonstrated in the linear mixed model investigation (Supplementary Table 1). The mean WMH volume in ‰ of ICV at baseline, and after one, three, and five years of the intervention in the Control group (in orange) and the combined supervised exercise group (MICT&HIIT) in blue. Abbreviations: WMH: white matter hyperintensities; ICV: intracranial volume; MICT: moderate-intensity continuous training; HIIT: high-intensity interval training.
