# Supplementary material for: Five years of exercise intervention at different intensities and development of white matter hyperintensities in community dwelling older adults, a Generation 100 sub-study
Source: Aging (Albany NY). 2022 Jan 18;14(2):596–622. doi: 10.18632/aging.203843 (PMC8833118; doi:10.18632/aging.203843)
Supplement: Supplementary Table 1 [file aging-14-203843-s002.pdf]

SUPPLEMENTARY TABLE

Supplementary Table 1. The effect of the interventions across the five-year intervention period on WMH, PWMH, DWMH and WM-hypo volumes (corrected for ICV) adjusted for age, sex, and baseline outcome values for the two intervention groups combined (MICT and HIIT) compared to the control group.

	WMH			PWMH.			DWMH.			WM-hypo.		
**Predictors**	**Est.**	**CI (95%)**	***p-value***	**Est.**	**CI (95%)**	***p-value***	**Est.**	**CI (95%)**	***p-value***	**Est.**	**CI (95%)**	***p-value***
Women	2.01	−1.21, 5.22	0.219	0.88	−1.07, 2.82	0.375	1.21	−0.34, 2.75	0.124	−0.79	−1.81, 0.24	0.130
Age	0.26	−0.56, 1.09	0.528	0.46	−0.04, 0.97	0.073	−0.17	−0.56, 0.23	0.404	0.29	0.02, 0.56	**0.035**
Year one	1.05	−0.68, 2.79	0.232	0.49	−0.67, 1.65	0.403	0.84	0.08, 1.60	**0.030**	0.01	−0.69, 0.70	0.983
Year three	2.40	−0.51, 5.30	0.105	1.21	−0.62, 3.04	0.193	1.30	−0.04, 2.63	0.057	0.20	−0.83, 1.23	0.701
Year five	7.67	3.13, 12.21	**0.001**	4.57	1.76, 7.38	**0.002**	3.07	0.95, 5.19	**0.005**	0.72	−0.81, 2.24	0.356
MICT&HIIT	−1.17	−4.39, 2.05	0.473	−0.97	−2.93, 1.00	0.331	−0.42	−1.96, 1.13	0.591	−0.52	−1.63, 0.61	0.363
MICT&HIIT*1-year	0.08	−1.66, 1.82	0.931	0.05	−1.19, 1.28	0.943	−0.15	−0.84, 0.55	0.680	0.31	−0.49, 1.11	0.444
MICT&HIIT*3-year	0.73	−1.06, 2.51	0.422	0.55	−0.71, 1.81	0.391	0.06	−0.66, 0.76	0.880	−0.05	−0.87, 0.77	0.897
MICT&HIIT*5-year	1.21	−0.61, 3.04	0.191	1.16	0.10, 2.22	**0.032**	−0.18	−0.91, 0.54	0.620	−0.44	−1.27, 0.39	0.300
AIC			2105.5			1819.4			1489.8			1519.7

There was a significant interaction between year and the exercise interventions combined (MICT and HIIT), this term was therefore kept for final analysis. Other non-significant interaction terms (between sex, age, or the exercise interventions combined [MICT&HIIT]) were removed from the statistical models. Abbreviations: WMH: white matter hyperintensities; PWMH: periventricular WMH; DWMH: deep WMH; WM-hypo: white matter hypointensities; ICV: intracranial volume; CI: confidence interval; MICT: moderate-intensity continuous training; HIIT: high-intensity interval training; MICT&HIIT: MICT and HIIT combined into one intervention group; Est.: estimates; AIC: Akaike’s information criterion.
